# Supplementary figures and images for: RNA polymerase III is involved in regulating Plasmodium falciparum virulence
Source: eLife. 2024 Jun 26;13:RP95879. doi: 10.7554/eLife.95879 (PMC11208047; doi:10.7554/eLife.95879)

1 cycle without Shield

+S    -S

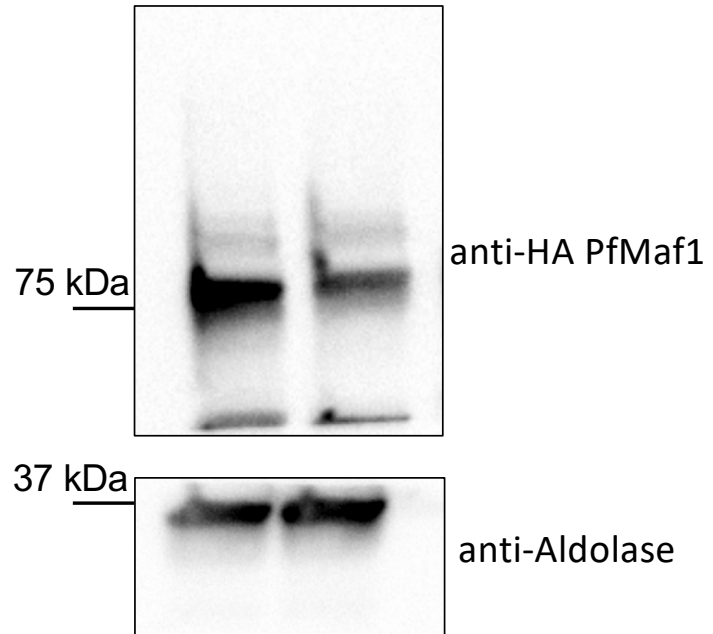

3 cycles without Shield

+S    -S

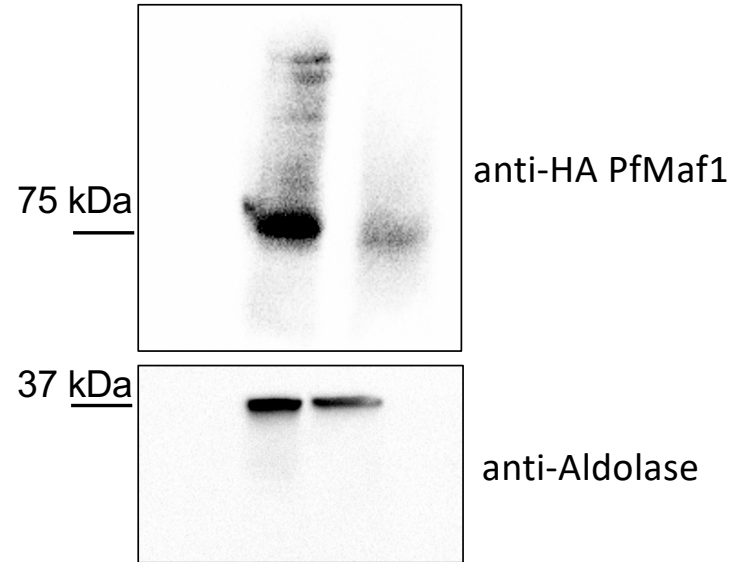

Supplement: Figure 3—source data 1. [file elife-95879-fig3-data1.pdf]

2 cycles without Shield

anti-Aldolase

+S -S

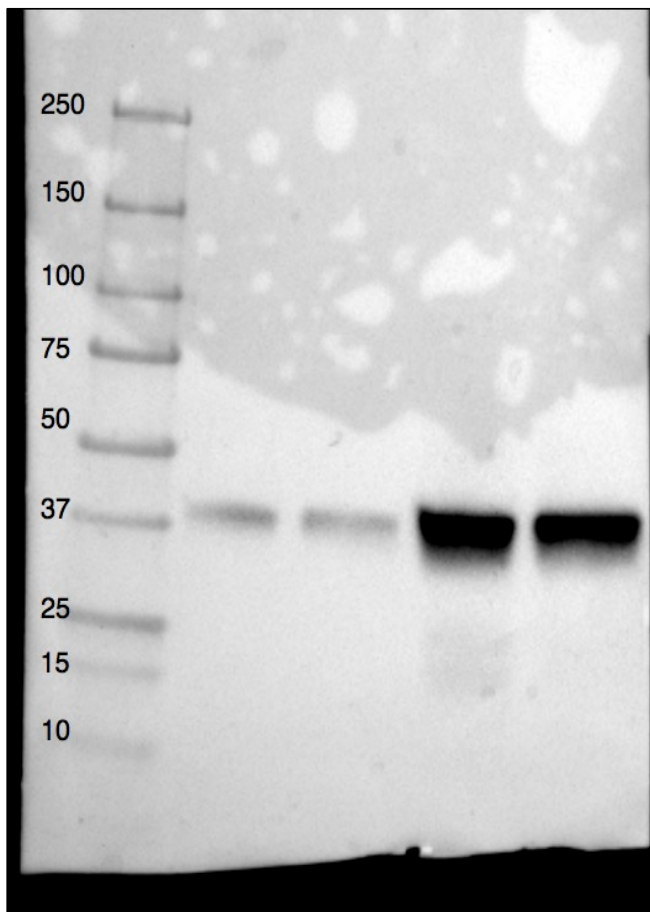

anti-HA PfMaf1

+S -S

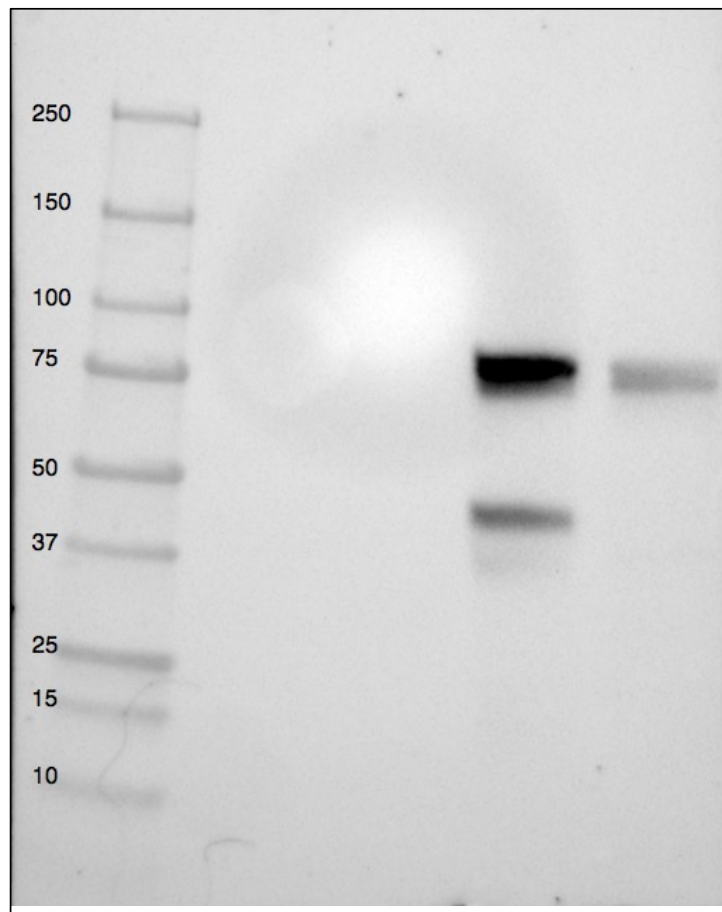

Supplement: Figure 3—source data 2. [file elife-95879-fig3-data2.pdf]

# IP-WB Maf1 +/- MgCl<sub>2</sub>

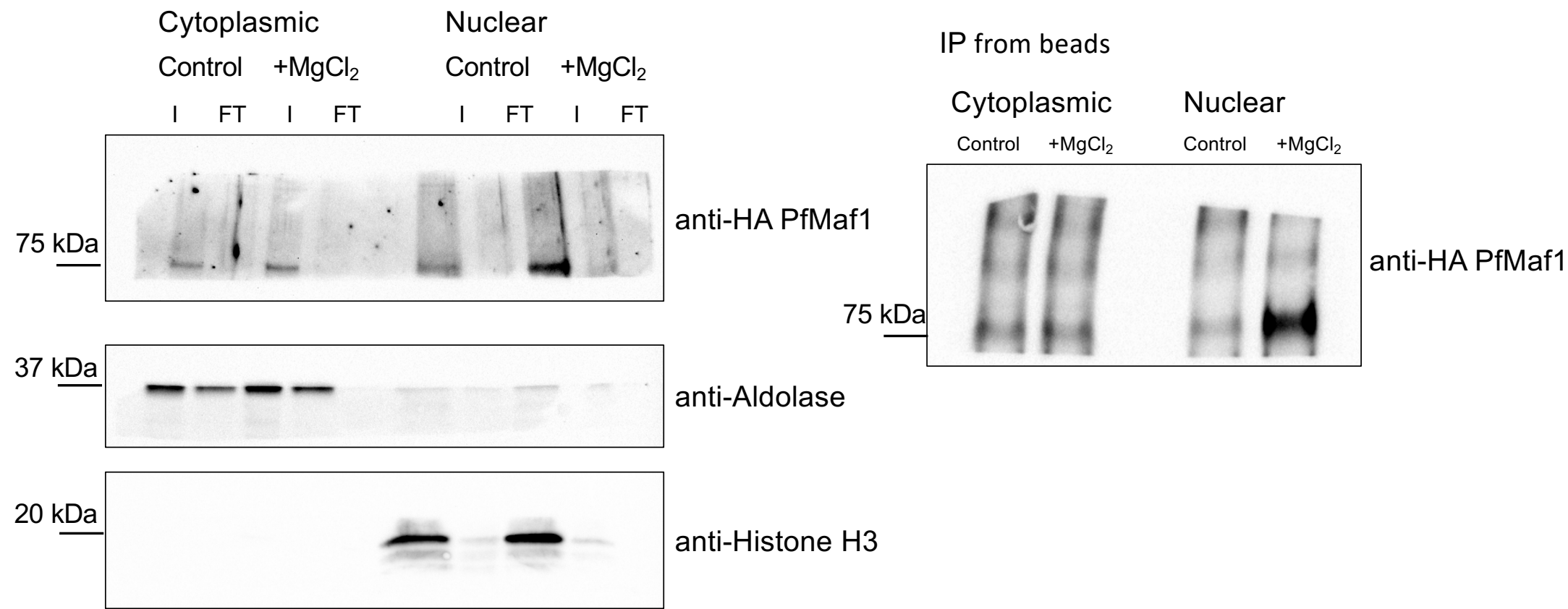

I: Input  
FT: Flow through

Supplement: Figure 4—source data 1. [file elife-95879-fig4-data1.pdf]

## Cytoadhesion binding assay membrane WB

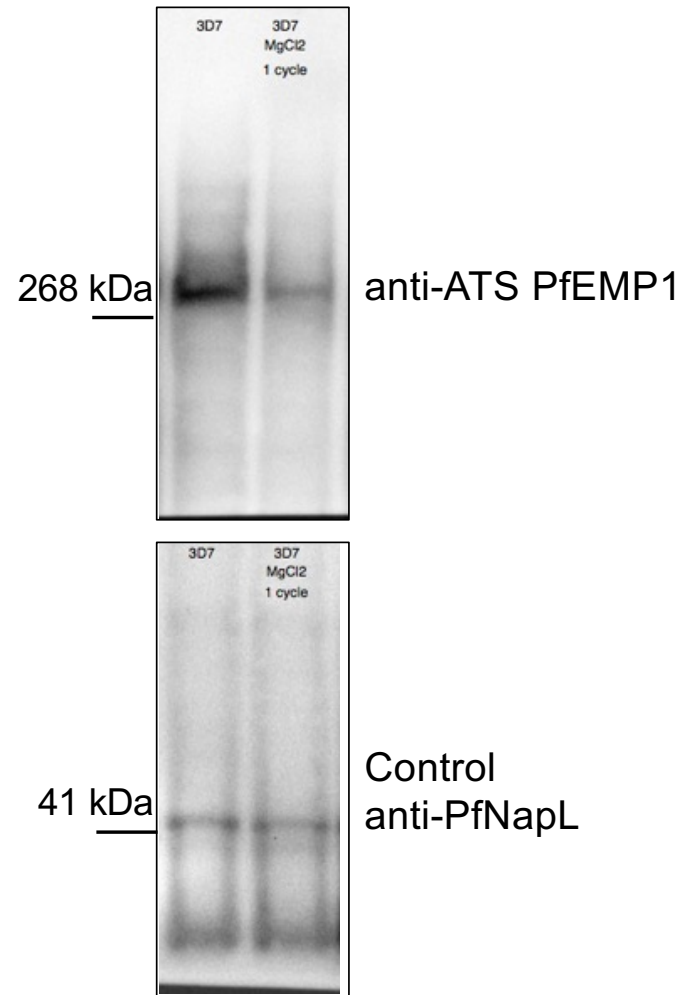

Supplement: Figure 4—source data 2. [file elife-95879-fig4-data2.pdf]
