# Supplementary material for: RNA polymerase III is involved in regulating Plasmodium falciparum virulence
Source: eLife. 2024 Jun 26;13:RP95879. doi: 10.7554/eLife.95879 (PMC11208047; doi:10.7554/eLife.95879)
Supplement: Supplementary file 1. — Primer pairs used are listed including the forward and reverse sequences. [file elife-95879-supp1.docx]

| **Primer pair** | **Forward** | **Reverse** |
| --- | --- | --- |
| Pool  RUF6 A  RUF6 B | 5’-AAGCTGCCTCAGTAGCCCA-3’ | 5’-AAAAATTGCGCCACCCCC-3’ |
|  | 5’-AAGCTGCCCCAGTAGCCCA-3’ | 5’-AAAAATTGCGCCGCCCCC-3’ |
| rRNA A1 (from (Mancio-Silva *et al*, 2010)) | 5’-TGTTTTCTTTTTTCTAAGTTT-3’ | 5’-TCACCTCATTTGAAGCAA-3’ |
| tRNA Alanine (PF3D7_0411500) | 5’-GGGCAGGTGGTGTAGTGG-3’ | 5’-TGCTGGACAGACGGGGAATT-3’ |
| tRNA Asparagine (PF3D7_0714700) | 5’-GCAAGTATTTCCGCCTGTCA-3’ | 5’-GAATTGAACCCGGGTCTTCC-3’ |
| tRNA Valine (Pf3D7_0312600) | 5’-GCGGGCATGGTCTAGTGG-3’ | 5’-ACTACGGGCACCGAGGATC-3’ |
| FBA fructose-bisphosphate aldolase (PF3D7_1444800) | 5’-TGTACCACCAGCCTTACCAG-3’ | 5’-TTCCTTGCCATGTGTTCAAT-3’ |
| UCE ubiquitin-conjugating enzyme (Pf3D_70812600) | 5’-TAACAGCCCAGCGAATCAAG-3’ | 5’-CGGCATCTTCTTCAGCTTTCTG-3’ |
| *var* gene (PF3D7_1240900) | 5’-CAAAATGGTAGTGATGGTGGTCG-3’ | 5’-CCCCCTGCTTTATTATCTTTCGTC-3’ |
| Pool  *var* DBLalpha | 5’-GCACGAACTTTTGCA-3’ | 5’-GCCCCATTCGTCGAACC-3’ |
|  | 5’-GCACGCAGTTTTGCA-3’ | 5’-GCCCCATTCCTCGAACC-3’ |

**Supplementary file 1. qPCR analysis primer pairs**

Primer pairs used are listed below including the forward and reverse sequences.
